# Trends in *Helicobacter pylori* resistance to clarithromycin: from phenotypic to genomic approaches

**DOI:** 10.1099/mgen.0.000344

**Published:** 2020-03-02

**Authors:** Andreia T. Marques, Jorge M. B. Vítor, Andrea Santos, Mónica Oleastro, Filipa F. Vale

**Affiliations:** ^1^​ Host–Pathogen Interactions Unit, Research Institute for Medicines (iMed-ULisboa), Faculty of Pharmacy, Universidade de Lisboa, Lisboa, Portugal; ^2^​ Department of Biochemistry and Human Biology, Faculty of Pharmacy, Universidade de Lisboa, 1649 003 Lisbon, Portugal; ^3^​ National Reference Laboratory for Gastrointestinal Infections, Department of Infectious Diseases, National Institute of Health Dr Ricardo Jorge, Lisbon, Portugal

**Keywords:** *Helicobacter pylori*, clarithromycin, resistance, 23S ribosomal RNA subunit, next-generation sequencing, point mutations

## Abstract

For a long time *Helicobacter pylori* infections have been treated using the macrolide antibiotic, clarithromycin. Clarithromycin resistance is increasing worldwide and is the most common cause of *H. pylori* treatment failure. Here we review the mechanisms of antibiotic resistance to clarithromycin, detailing the individual and combinations of point mutations found in the 23S rRNA gene associated with resistance. Additionally, we consider the methods used to detect clarithromycin resistance, emphasizing the use of high-throughput next-generation sequencing methods, which were applied to 17 newly sequenced pairs of *H. pylori* strains isolated from the antrum and corpus of a recent colonized paediatric population. This set of isolates was composed of six pairs of resistant strains whose phenotype was associated with two point mutations found in the 23S rRNA gene: A2142C and A2143G. Other point mutations were found simultaneously in the same gene, but, according to our results, it is unlikely that they contribute to resistance. Further, among susceptible isolates, genomic variations compatible with mutations previously associated with clarithromycin resistance were detected. Exposure to clarithromycin may select low-frequency variants, resulting in a progressive increase in the resistance rate due to selection pressure.

## Data Summary

The genome accession numbers and metadata are presented in the Repositories section and in Table 1.

**Table 1.** Association between point mutations in the 23S rRNA and the clarithromycin-resistant phenotype of 17 pairs of *H. pylori* isolates from the antrum and corpus

Genome	Phenotype	Position of the mutation	Clarithromycin MIC (mg l^−1^)
10 087A	Resistant	A2143G	8
10087C	Resistant	A2143G	8
10 103A	Resistant	A2142C, G2212A	>256
10103C	Resistant	A2142C, G2212A	>256
10 120A	Resistant	A2143G, C2759T	12
10120C	Resistant	A2143G, C2759T	12
10 198A	Resistant	A2143G, C2772T	24
10198C	Resistant	A2143G, C2772T	24
10 211A	Resistant	A2143G	12
10211C	Resistant	A2143G	12
10 212A	Resistant	A2143G	2
10212C	Resistant	A2143G	2
10 104A	Susceptible	–	–
10104C	Susceptible	–	–
10 127A	Susceptible	T2182C	–
10127C	Susceptible	T2182C	–
10 128A	Susceptible	–	–
10128C	Susceptible	–	–
10 133A	Susceptible	–	–
10133C	Susceptible	–	–
10 144A	Susceptible	–	–
10144C	Susceptible	–	–
10 147A	Susceptible*	–	–
10147C	Susceptible*	–	–
10 201A	Susceptible	–	–
10201C	Susceptible	–	–
10 210A	Susceptible	–	–
10210C	Susceptible	–	–
10 215A	Susceptible	–	–
10215C	Susceptible	–	–
10 218A	Susceptible	–	–
10218C	Susceptible	–	–
10 222A	Susceptible	–	–
10222C	Susceptible	–	–

*With resistant clones.

A, antrum isolate; C, corpus isolate.

**Impact Statement**

Antibiotic-resistant *Helicobacter pylori* strains are increasing in prevalence, as acknowledged recently by the World Health Organization when *H. pylori* was included in a list of bacteria that pose the greatest threat to human health. We reviewed the mechanisms of resistance to clarithromycin, producing a complete compendium of all mutations found in the 23S rRNA gene that have been associated with resistance to this macrolide antibiotic. Additionally, we described the phenotypic and genotypic methods used for detection of clarithromycin resistance, placing particular emphasis on the usefulness of whole-genome sequencing in detecting the resistance and progression towards resistance found in minority genomic variants.

## Introduction

*Helicobacter pylori* is a long-lasting human (stomach) traveller companion causing gastritis, peptic ulcer and gastric carcinoma [1, 2]. Clarithromycin (CLA) has been the basis for *H. pylori* treatment because of its low minimal inhibitory concentration (MIC), good mucosal diffusion and relatively small effect on gastric acidity [3]. For years, this treatment consisted of triple therapy that combined CLA with either amoxicillin or metronidazole, and a proton pump inhibitor (PPI) [4]. However, the efficacy of triple therapy is in decline, mostly due to *H. pylori* resistance to CLA, contributing to the increasing burden of multidrug-resistant Gram-negative infection. This global antibiotic crisis was recognized by the World Health Organization (WHO), who in 2017 published a list of antibiotic-resistant priority pathogens for research and development of new antibiotics, including CLA-resistant *H. pylori* [5].

CLA is a macrolide derived from erythromycin, whose bacteriostatic activity depends on its ability to inhibit the bacterial protein synthesis [6]. Protein synthesis is critical to life and is performed in a very old nanomachine, the ribosome. This nanomachine arose from the molecular evolution that predated the first living cells [7, 8]. Ribosomes have two subunits, the large and the small, made of rRNA and proteins, presenting basic machinery to synthetize polypeptides: both subunits join, embracing an mRNA molecule, after recognition of a starter sequence, and both have a similar site to receive/dock the tRNA, and finally a similar exiting tunnel for the nascent protein chain [9]. But, as they are very old, enough time has passed for the evolution of mechanisms and compounds to stall this crucial nanomachine. Organisms who had that ability in starvation times could stop others from consuming resources that are fundamental for their own survival. Accordingly, there are several molecules that target different parts of the ribosome, all of them produced by bacteria, such as chloramphenicol, tetracyclines, streptomycin and erythromycin [10].

**Fig. 1.**
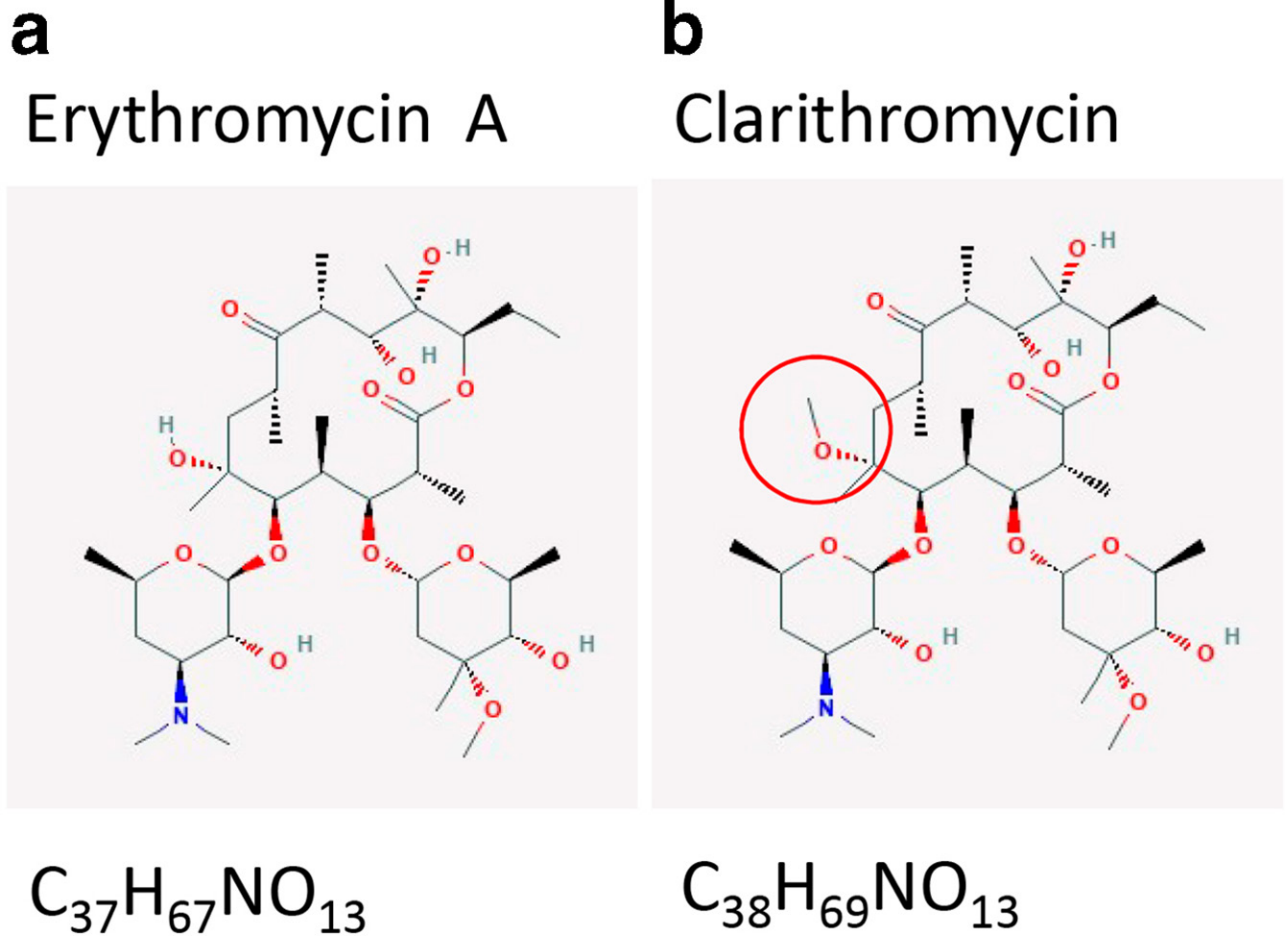
Two-dimensional chemical structure of erythromycin A (a) and CLA (b). CLA is the 6-O-methyl ether oferythromycin A. Structures from PubChem (accessed August 2019) [67].

Erythromycin is a natural antibiotic produced by *Saccharopolyspora erythraea* (formerly *Streptomyces erythraeus *) whose original strain produced four types of molecules: erythromycin A, B, C and D [11]. Erythromycin A is a macrolide (a macrocyclic lactone with a ring of 12 or more members derived from a polyketide) that inhibits bacterial protein synthesis by interfering with aminoacyl translocation, preventing the transfer of the tRNA bound at the A site of the rRNA complex to the P site of the rRNA complex (Fig. 1a) [12]. Erythromycin may be bacteriostatic or bactericidal, depending on the organism and drug concentration. However, the action of macrolides is not simple. A recent review points to other important macrolide actions, such as being modulators of peptide bond formation, not only ribosome tunnel plugs [10].

This review is about *H. pylori* resistance to a small man-made alteration of erythromycin: the 6-O-methyl ether of erythromycin A, named clarithromycin (CLA) (Fig. 1B). CLA inhibits bacterial protein synthesis by reversibly binding to the 50S ribosomal subunit [6]. Its mechanism of action is to bind to the peptidyl transferase loop of the V domain of 23S ribosomal RNA (23S rRNA) gene, which results in structural changes and dissociation of peptidyl-tRNA from ribosome, interfering with nascent peptide chain elongation [13]. Misuse of antibiotics accelerates resistance, namely when targeting ribosome, which is very flexible and a minor conformational change could be enough to avoid the action of natural or semi-synthetic antibiotics [14]. Bacterial populations have a variability of rRNA sequences that assures the survival of some of its members, which we will show using our own results.

### CLA resistance mechanism

*H. pylori *’s antimicrobial resistance is mainly acquired by point mutations, which are transmitted vertically by binary fission, resulting in a progressive increase in the resistance rate due to selection pressure.

Several PCR-based studies have demonstrated that point mutations in the peptidyl transferase loop of the V domain of 23S rRNA gene are responsible for the CLA resistance phenotype in clinical *H. pylori* strains from various geographical locations [15–20]. These mutations are able to disrupt the peptidyl transferase loop conformation and inhibit the binding between CLA and the 23S rRNA, reducing its efficiency and leading to a resistance phenotype [18].

Two copies of the 23S rRNA operon are present in the *H. pylori* genome [19, 21] and, for most *H. pylori* strains, mutations are generally found in both copies; nevertheless, a heterozygote phenotype is sufficient to confer intermediate resistance to CLA [16, 22]. Resistant strains can be divided into two groups: a high level of resistance (MIC >64 mg l^−1^) and a low level of resistance (0.5≤MIC≤1 mg l^−1^).

**Fig. 2.**
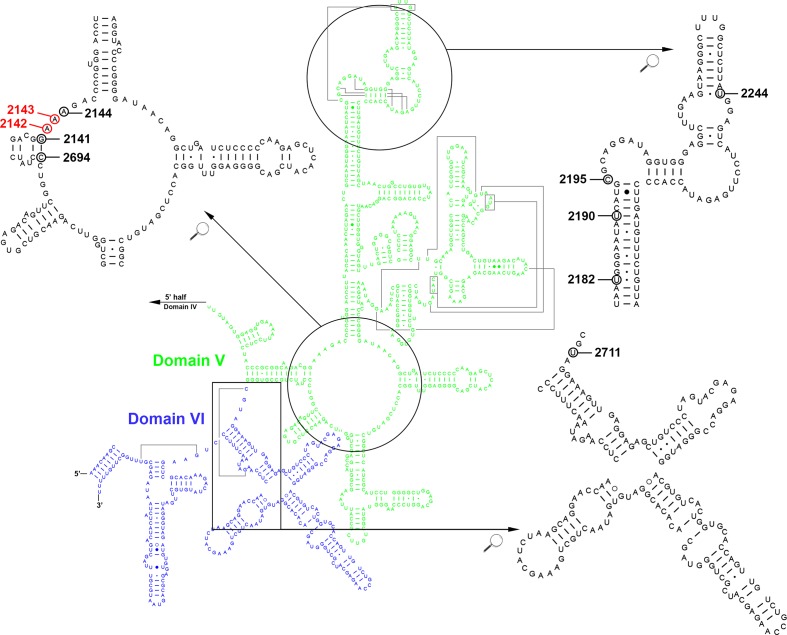
Secondary structure model of the peptidyl transferase centre in the domain V (green) and domain VI (blue) of the *H. pylori* 23S rRNA. The indicated point mutation positions correspond to single mutations (not requiring combination with other mutations) conferring low-level to high-level CLA resistance. Mutations 2141 (G2141A), 2144 (A2144T), 2182 (T2182C), 2190 (T2190C), 2195 (C2195T), 2244 (T2244C), 2694 (C2694A) and 2711 (T2711C) are indicated by black circles and the most prevalent mutations 2142 (A2142G) and 2143 (A2143G) are indicated by red circles. Image available at (and modified from) http://www.rna.icmb.utexas.edu (accessed July 2019) [68]). The circled nucleotides indicate the positions of mutations that confer CLA resistance in *H. pylori* (details and references are given in Table 2).

The most prevalent and well-documented mutations in *H. pylori* occur in two specific adjacent nucleotide positions, an adenine-to-guanine transition at either position 2142 (A2142G) or 2143 (A2143G), or, less frequently, an adenine-to-cytosine transversion at position 2142 (A2142C), and these mutational events are responsible for more than 90 % of CLA resistance in developed countries [23]. In particular, mutation at position 2143 is usually associated with different levels of resistance (MICs ranging from 2 to 256 mg l^−1^), while strains with mutation at position 2142 frequently exhibit a more restricted resistance (MIC of 64 mg l^−1^). These two mutations (2142 and 2143) were originally described as positions 2058 and 2059, based on *Escherichia coli* 23S rRNA sequence [15, 16, 18, 24] and later changed to 2143 and 2144 according to the *H. pylori* 23S rRNA sequence GenBank U27270 [20]. After Taylor *et al*. determined the DNA sequences of the two copies of the 23S rRNA gene from *H. pylori* strain UA802 and compared the sequences from CLA-resistant strains, they proposed that he positions associated with CLA resistance were nucleotides 2142 and 2143 [19] and most investigators choose to use this nomenclature. Furthermore, it has been reported that other mutations are able to confer CLA resistance, including mutations A2115G, G2141A, A2144T and T2289C [25–28], whilst C2694A and T2717C have been associated with low resistance levels [29, 30]. Fig. 2 illustrates point mutations that confer CLA resistance in the model of the 23S rRNA domains V and VI. The conversion T2182C is one controversial mutation and has been reported as not being required for CLA resistance, as well as conferring low-level to high-level resistance, with an MIC >64 mg l^−1^ [30–33]. Other mutations have been described in the literature (i.e. G1939A, C2147G, G2172T, T2215C and C2245T), but their role in failure of CLA-based therapy is still not proven or is not being consistently reported [34–38]. Table 2 summarizes a complete list with described single and combined point mutations conferring CLA resistance (search carried out in July 2019, using the keywords CLA resistance, *H. pylori *, 23S rRNA and efflux pumps, and a ‘snowball’ search – pursuing references of references).

**Table 2.** Point mutations in the 23S rRNA region and MIC values associated with CLA-resistant *H. pylori* strains

Position*	Mutation	MIC (mg l^−1^)	References
**1592†**	A1592G+T2182C	1	[69]
	A1592G+C2195T	2	[69]
	A1592G + T1644C + A1821G + G1826A + T1830C+T2182C	1	[69]
**1592†**	A1592T + A1821G + G1826A + T1830C+T2182C	4	[69]
**1652†**	A1652G	2	[69]
**1694†**	G1694A + A1738G + C1953T	4	[69]
**1738†**	A1738G + G1826C+C2195T	1	[69]
**1769†**	C1769T	1	[69]
**1821**	A1821G+T2182C	1	[31]
	A1821G + G1826A + T1830C+T2182C	4	[31]
**1826†**	G1826A+A2143G	4	[69]
**1939**	G1939A + T1942C+A2142G+C2147G	1	[35]
	G1939A+A2306G	0.064 to 0.5	[34]
**1944**	C1944T+G2212A	0.064 to 0.5	[34]
**1953**	C1953T+A2143G+T2182C+A2223G+T2244C	1.5 to 256	[70]
**2115**	A2115G	1.24 to 4	[25, 26]
	A2115G+A2141G	4	[22]
	A2115G+A2142G	4	[26]
	A2115G+A2143G	4	[26]
	A2115G+A2144T	4	[26]
**2141**	G2141A	1.25 to 28	[25–27]
**2142**	A2142C	64 to >256	[15, 27, 71–73]
	A2142C+A2142G	na	[73]
	A2142C+A2143G	na	[71]
	A2142C+G2212A	>256	This work
	A2142C+A2142G+A2143G	na	[71]
**2142**	A2142G	0.1 to 256	[16, 22, 26, 27, 35, 38, 72–76]
	A2142G+A2143G	4	[26, 71, 73]
	A2142G+A2144T	na	[71]
	A2142G+T2182C	1 to 256	[76, 77]
	A2142G+C2195T	≤32	[74]
	A2142G+A2223G	>32	[74]
	A2142G+A2143G+A2144T	256	[26]
	A2142G+T2182C+C2195T	>32	[74]
**2143**	A2143G	0.1 to 256	[16, 22, 26, 27, 32, 35, 38, 71–76, 78]
	A2143G+A2174G	≤32	[74]
	A2143G+T2182C	0.5 to 45	[36, 74, 76–78]
	A2143G+A2223G	≤32	[74]
	A2143G+G2224A	4	[27]
	A2143G+T2244C	3 to 48	[70]
	A2143G+C2245T	16	[27]
	A2143G+C2759T	12	This work
	A2143G+C2772T	24	This work
	A2143G+T2182C+T2190C	52	[78]
	A2143G+T2182C+C2195T	53.3	[78]
	A2143G+T2182C+A2223G	80	[78]
	A2143G+T2182C+T2244C	1.5 to 96	[70]
	A2143G+C2195T+A2223G	≤32	[74]
	A2143G+A2223G+T2244C	1.5 to 96	[70]
	A2143G+T2182C+A2223G+T2244C	1.5 to 256	[70]
	A2143G+T2182C+T2244C+A2302G	1.5 to 256	[70]
**2143**	A2143T+T2182C+G2172T+G2254T	na	[36]
**2144**	A2144T	3 to 134	[25–27]
**2182**	T2182C	1 to >64	[31, 32, 72, 74, 76, 78]
	T2182C+T2244C	3 to 48	[70]
	T2182C+T2190C+C2694A	>0.5	[30]
**2190**	T2190C	na	[79]
**2195**	C2195T	na	[79]
	C2195T+T2182C+A2223G+T2244C	1.5 to 256	[70]
**2224**	G2224A+T2289C	64	[28]
	G2224A+C2245T+T2289C	>256	[28]
**2244‡**	T2244C	1.5 to 5	[70, 80]
**2289**	T2289C	8	[28]
**2694**	C2694A	1	[30]
**2711§**	T2711C	0.5 to 1	[29]

na, not available.

*Consolidated position according the nomenclature published by Taylor *et al*.[19] (1997), which corresponds to the reference sequence *H. pylori* 23S rRNA gene (GenBank: U27270.1) nucleotide.

†Matta *et al*. [69] describe these positions as 1593, 1653, 1695, 1739, 1770 and 1827, respectively (reference sequence GenBank: U27270.1).

‡Khademi *et al*. [80] describe this position as 2243.

§Fontana *et al*. [29] describe this position as 2717.

### Other resistance mechanisms

Another possible mechanism for CLA resistance is multidrug efflux pump systems. Efflux of antimicrobial compounds is commonly observed in bacteria, reducing intracellular antimicrobial concentration [39, 40]. Efflux pumps of the resistance-nodulation-cell division (RND) family are responsible for macrolide resistance in Gram-negative bacteria and this mechanism has also been proposed for *H. pylori* [41, 42].

Three putative operons have been described as an RND efflux system in *H. pylori *: *hefABC* (hp0605–hp0607), *hefDEF* (hp0969–hp0971, recently denominated *cznABC*) and *hefGHI* (hp1327–hp1329, recently denominated *czcAB-crdB*), with the *hefABC* operon being most similar to multidrug efflux pumps. The *hefA*, *hefD* and *hefG* genes encode an outer-membrane protein TolC homologue of *E. coli *, while *hefB*/*hefC*, *hefE*/*hefF* and *hefH*/*hefI* genes are homologues of the *acrA*/*acrB* genes, encoding a membrane fusion and RND cytoplasmic pump proteins, respectively [39, 43, 44]. A fourth gene cluster including ORF hp1489–1487 was described, where hp1489 is a TolC homologue and hp1488 shows similarities with the *acrA* gene [45]. In *H. pylori* a synergistic effect between 23S rRNA mutations and efflux pumps is likely to be present in resistant strains; the former lowers the CLA affinity to the ribosome and the latter excretes the antibiotic. In some CLA-resistant strains presenting 23S rRNA mutations, it was shown that the presence of efflux pumps inhibitors (EPIs) was able to decrease the MIC of most of the studied strains by fourfold. The EPIs lead to an augmented intracellular CLA concentration, which binds to the ribosome even in the presence of mutations. However, in these cases, the final MICs were still in the resistant range [46]. Examination of the genetic variants of these four efflux pumps revealed that CLA resistant strains are more prone to single-nucleotide variants in all four clusters of efflux genes, with significant differences for cluster *hefABC* [47]. Although the exact underlying mechanism is unclear, it may involve antibiotic efflux. To the best of our knowledge, the efflux pumps alone (either by mutant variants or by differential expression profile) have not been associated with *H. pylori* CLA resistance.

Other novel candidates for CLA resistance likely present a synergistic effect with 23S rRNA point mutations. Indeed, after the exposure of a susceptible strain to low doses of CLA, mutations in genes *infB* (translation initiation factor IF-2) and *rpl22* (ribosomal protein L22) conferred low-level resistance to CLA (low MIC value), while mutations in these genes plus mutations in 23S rRNA increased the MIC value. Additionally, comparative proteomics analysis highlighted the possible involvement of outer-membrane proteins in CLA resistance. Resistant strains in comparison to susceptible strains presented upregulated UreaseB subunit and EF-Tu (elongation factor thermo unstable) and downregulated HofC (efflux pump) and OMP31 [48]. Finally, the *spoT* [bifunctional (p)ppGpp synthase] gene is involved in tolerance to CLA, upregulating transporter genes (HP0939, HP1017, HP0497 and HP0471) [49]. Overall, the mechanisms besides mutations in 23S rRNA appear to potentiate the effect of ribosomal mutations by interfering either with other translation machinery or with antibiotic transport, reducing its intracellular concentration.

### Detection methods

Antimicrobial susceptibility testing should be performed whenever possible to guide therapy selection. For CLA, for which the *in vitro* resistance is predictive of unsuccessful treatments, this test assumes particular relevance in the management of *H. pylori* infection, especially in high primary resistance regions [50]. Phenotypic and genotypic methods can be used to test susceptibility to CLA. Several phenotypic methods have been developed, such as the agar dilution method, which is considered to be the reference method in comparison to other techniques. The MIC breakpoint for CLA, which is based on epidemiological cut-off values, is 0.25 mg l^−1^ for susceptible and 0.5 mg l^−1^ for resistant strains [51]. This method is very time-consuming and is rarely performed in routine laboratories. The E-test method, based on gradient diffusion, with the ability to produce an MIC result, is currently the method of choice in most of the clinical laboratories performing antimicrobial susceptibility testing of *H. pylori *, since is adapted to slow-growing bacteria. A good correlation has been found between this method and the agar dilution method [51]. Breakpoint susceptibility testing is a simplified version of the agar dilution method. It consists of inoculating a line of the strain to be tested on an agar plate containing an antibiotic concentration equal to the breakpoint concentration that defines resistance. The broth dilution method is seldom used due to the difficulty of growing *H. pylori* in broth media. However, it is possible to use it and obtain acceptable MIC results if the broth is supplemented with serum or defibrinated blood [52–54]. Finally, the simplest and most economical method for routine susceptibility testing is the disk diffusion method, which is generally not recommended for slow-growing bacteria, although it has been validated to detect macrolide resistance accurately in *H. pylori *, while erythromycin is the recommended antibiotic for macrolide susceptibility testing.

In *H. pylori *, given the low number of chromosomal point mutations conferring CLA resistance (Table 2), accurate genotypic methods have developed exponentially. These tests are faster than the phenotypic methods, and are easy to establish in routine practice. The most common is based on amplification of the 23S rRNA by PCR, followed by different detection methods. PCR-RFLP was one of the first methods to be developed, making use of three different restriction enzymes, one for each of the three most common point mutations (A2142/3G and A2142C) [55]. This laborious method, based on a profile band resolved in an agarose gel, was rapidly replaced by faster and more sensitive methods, such as real-time PCR, which allows the detection of *H. pylori* as well as CLA resistance-associated point mutations in a single reaction. This test is usually based on a biprobe and fluorescence resonance energy transfer (FRET), allowing easy discrimination of the three mutations by melting curve analysis, with the potential of being used directly on gastric biopsies or on stool specimens, increasing the sensitivity of the method to detect mixed susceptible and resistant populations in a single sample [56]. Other PCR-based methods can vary in their detection method, for example combining multiplex conventional PCR and amplicon detection via a reverse hybridization and alkaline phosphatase reaction on a membrane strip coated with highly specific probes complementary to the selectively amplified nucleic acid sequences [57, 58]. Using PCR-based molecular methods on non-invasive samples such as stools makes susceptibility testing easier and more practical to perform, and therefore such methods are widely available. Genotypic methods not involving DNA amplification have also been developed, using fluorescence *in situ* hybridization (FISH), which is based on probes that hybridize with specific rRNA sequences of micro-organisms. The best performance is achieved using peptide nucleic acid probes, which are usually smaller than typical DNA probes, increasing their ability to penetrate the bacterial cell wall, and are more resistant to degradation by nucleases and proteases [59].

Finally, the development of high-throughput next-generation sequencing (NGS) methods has allowed the use of whole-genome sequencing as a genome-based typing method, additionally enabling antibiotic resistance determinants to be inferred. Table 3 presents an overview of some of the genotypic methods used to detect macrolide resistance in *H. pylori *.

**Table 3.** Genotypic methods used to detect macrolide resistance in *H. pylori
*

Based on 23S rRNA gene	Method	Reference
With amplification	RFLP	[55]
	PCR followed by reverse hybridization	[57, 58]
	Oligonucleotide ligation assay	[81]
	DNA enzyme immunoassay	[82, 83]
	Preferential homoduplex formation assay	[84]
	Real-time PCR	[56, 85]
	3’-mismatched reverse primer PCR	[86]
	Microelectronic chip array	[87]
	Dual-priming oligonucleotide‐based multiplex PCR	[88]
	Microarray	[89]
	Droplet digital PCR	[90]
Without amplification	Peptide nucleic acid-fluorescence *in situ* hybridization	[59, 91]
	Next-generation sequencing	[92]

### NGS for detecting resistance: an example

As previously stated, it has been reported that other mutations are able to confer CLA resistance, but their role is still controversial. We therefore used a set of antrum and corpus pairs of *H. pylori* clinical isolates in order to clarify the role of these less common mutations, as well as the power of NGS to detect resistance. The genomes of 17 pairs of isolates from a pool of colonies from the antrum and corpus of a paediatric population were sequenced, Illumina MiSeq assembled with SPAdes3.13 [60] and analysed. Antimicrobial susceptibility testing was performed by disk diffusion for erythromycin, and the E-test was used to determine the MIC for CLA for the resistant strains. Six pairs of isolates were CLA-resistant (12/34 isolates) and a total of six point mutations were detected (Table 1). All pairs of CLA-resistant isolates of antrum and corpus of the same patient displayed the same mutations. All these isolates have mutations at positions 2142 and 2143, with A2143G (83.3 % of isolates, 10/12) being predominant, followed by A2142C (16.7 % of isolates, 2/12). In detail, three patients had the single mutation A2143G for both antrum and corpus isolates (6/12 isolates), and three patients had double mutations, A2142C+G2212A for one case (2/12 isolates), A2143G+C2759T for the second case (2/12 isolates) and A2143G+C2772T for the last case (2/12 isolates). The MICs for CLA varied between 2 and 24 mg l^−1^ for isolates harbouring the mutation A2143G, while the MIC was >256 mg l^−1^ for the two isolates from the same patient with the mutation A2142C (Table 1). These values are in the range of those described extensively in the literature for these mutations, making it difficult to predict the role of the remaining mutations found simultaneously.

Two additional mutations found in combination with A2143G, C2759T and C2772T, both located at 23S rRNA domain VI, are reported for the first time (Fig. 2 and Table 2). A T2182C mutation was found in two susceptible isolates (antrum and corpus from same patient), which is in agreement with its controversial role in CLA resistance [30–33].

Based on the results from our setting, we conclude that the resistance phenotype is related to the presence of the well-described 2142 and 2143 point mutations, while the presence of the other reported mutations *per se* cannot confer a resistance phenotype, corroborating previous studies. Regarding the prediction of resistance levels, we cannot make a conclusive statement about the role of these less common or new mutations, since the MICs described are all in the range of the previously described values for isolates harbouring the well-described 2142 and 2143 point mutations. Nevertheless, this example of the application of NGS for CLA resistance determination shows the power of genome sequencing for correctly detecting resistance in clinical strains. In fact, all of the cases with MIC values that were compatible with resistance to CLA presented the most common mutations in the 23S rRNA gene associated with resistance (Table 1). The continuous reduction of the cost of genome sequencing, coupled with the multi-tasking performance of NGS (e.g. it is also useful for molecular typing, population genetics or epidemiology), makes this technique a strong candidate for introduction into clinical laboratories for the testing of antibiotic resistance.

### Genomic variability of the 23S rRNA gene: genome-based prediction of evolution towards CLA resistance

*H. pylori* genomes are highly diverse and not infrequently strains infecting the same host are genetically differentiated [1, 61]. Moreover, *H. pylori* genomes evolve rapidly during chronic infection [62, 63] and laboratory culture [64], evidencing its remarkable adaptive capacity to its niche. The genomes available for each bacterial strain are typically the canonical genomes, i.e. the most frequent residues of nucleotides at each position. The variation within a bacterial genome is commonly disregarded so that a consensus working sequence is available and also due to the difficulty of ascertaining if it represents a true variation or a sequencing error [64]. Importantly, the 23S rRNA gene is present in two copies in the *H. pylori* genome, which due to their similarity are assembled in the same *locus*. In fact, a common cause of the underestimation of gene number is the collapse of gene copies into a single locus that due to highly similarity in sequence are challenging to assemble as separate loci [65]. To understand the diversity within *H. pylori* clinical isolates, we have worked with the same set of *H. pylori* strains and looked for genomic variants in the 23S rRNA gene presenting a coverage level >100×, and at least 10 copies of the variant. Sequence reads were mapped to a canonical genome and a BAM file was produced using SAMtools [66]. Variant calling over BAM files was performed with Geneious 8.1.9, selecting variants at a frequency variant >1 % and maximum variant *P*-value 10E-6. Two out of 11 pairs of strains (18.1%) phenotypically susceptible to CLA presented variants matching mutations associated with CLA resistance (Table 4). Exposure to CLA may select low-frequency variants that lead to therapeutic failure and the emergence of a resistant strain. This appeared to be the case for the pair 10 147A/10147C, which is susceptible to CLA but presented a few colonies growing at high CLA concentration (Tables 1 and 4), suggesting a genotype evolving towards CLA resistance. For one of the pairs of resistant isolates (10 103A and 10103C), a genomic variation was also observable: most of the reads had the mutation A2142C, but the mutation A2142G was also found with a frequency of 28.5 and 28.2 %, respectively (Table 1). Both mutations are associated with CLA resistance.

**Table 4.** Genomes of *Helicobacter pylori* strains susceptible to CLA presenting variants matching described mutations in 23S rRNA for CLA resistance

Genome	Position	Change	Variant frequency (%)	Coverage
10 222A	2142	A -> G	6.3	191
10222C	2142	A -> G	6.9	275
10 147A	2142 *	A -> G	2.5	204
	2301	A -> G	4.7	235
10147C	2142	A -> G	12.9	240
	2301	A -> G	12.0	259

*Although fewer than 10 reads were observed with mutation A -> G, the result is presented because of the consistency between antrum and corpus isolates.

High-depth whole-genome sequencing is thus a powerful technique not only to determine the mutation associated with CLA resistance, but also to predict the evolution inferred by variant call analysis, examining the depth of reads mapped to each nucleotide variation position known to be associated with resistance. In addition, although the correlation between CLA resistance detected by phenotypic and genotypic methods is in general good, the latter is much more accurate in detecting low numbers of mutated bacterial cells within a pool of otherwise susceptible bacteria. According to our data, the genomic variability showed that about one fifth of the susceptible isolates coming from a pool of colonies is likely to have experienced microevolution events, i.e. share a common ancestor and present distinct genomic alterations within patient. In these cases, CLA may act as a selective agent of the minority and resistant variants. Therefore, the introduction of NGS for CLA resistance testing is additionally important as in addition to identifying strains that are resistant, it may also contribute to the identification of variants in which the resistant genotype is in minority, avoiding the selection of this resistant subpopulation.

### Conclusion

The resistance mechanism of CLA is mainly inscribed on three mutations found on the 23S rRNA gene, impairing target recognition by the antibiotic. In this study, mutations A2142C and A2143G were also detected in the 23S rRNA gene V domain of CLA-resistant *H. pylori *. Mutation T2182C was found in susceptible isolates. Several methods allow the detection of CLA resistance. NGS not only allows us to verify the presence of mutations in canonical genomes associated with resistance, but is also useful to ascertain evolution towards resistance evidenced by genomic variation matching known mutations associated with CLA resistance. There will be always antibiotic resistance to old and new molecules, because that is how bacteria had survived for millions of years. Therefore, the strategy to overcome CLA resistance should be to continue to study *H. pylori* biology to find new targets to eliminate the bacteria and to design new molecules.

## Data Bibliography

1. National Institute of Health, Portugal. Genbank, SRR9930173–SRR9930179 (2020).
